# Structural Characteristics Analysis of *Pinus taiwanensis* Plantation in Climate Transition Zone

**DOI:** 10.3390/plants15121842

**Published:** 2026-06-14

**Authors:** Mengli Zhou, Jianbo Shen, Peilin Pang, Fang Guo, Dongfeng Yan

**Affiliations:** 1 College of Forestry, Henan Agricultural University, Zhengzhou 450046, China; mlzhou@henau.edu.cn (M.Z.); pangpeilin2258@163.com (P.P.); guofang1979@henau.edu.cn (F.G.); 2 Wenzhou Key Laboratory of AI Agents for Agriculture, Wenzhou Vocational College of Science and Technology, Wenzhou 325006, China; lyshenjianbo@163.com

**Keywords:** *Pinus taiwanensis* plantation, stand spatial pattern, diameter distribution structure, density gradient, analytic hierarchy process-entropy weight method

## Abstract

Understanding the structural characteristics of *Pinus taiwanensis* plantations in climatically transitional regions is essential for developing science-based management strategies under global change. This study investigated 23 plots in Huangbai Mountain Forest Farm, Henan Province, China, classified into low-, medium-, and high-density stands (*n* = 9, 9, and 5, respectively). Diameter distributions were fitted using six probability functions, and four spatial structure parameters—mixing degree (*Mc*), size ratio (*U*), uniform angle index (*W*), and forest layer index (*S*)—were quantified. In addition, five comprehensive spatial structure indices—average superiority coefficient index (*SPV*), spatial structure comprehensive index (*Q*), stand spatial structure distance index (*FSI*), Comprehensive Distance Evaluation (*CDEV*), and Comprehensive Assessment of Proximity Vector (*CAPV*)—were constructed using a combined analytic hierarchy process and entropy weight method. Given the unbalanced sample sizes, non-parametric Kruskal–Wallis tests were employed for comparisons, and bootstrap resampling (1000 iterations) was performed to assess the reliability of mean estimates. The results showed that both the Gamma and Weibull distributions were equally suitable for describing diameter distribution under different stand densities, as their *AIC* differences were below 2 for all density classes. Correlation analysis indicated that the relative importance of spatial parameters followed the order *S* > *U* > *Mc* > *W*. Medium-density stands exhibited the most optimal spatial structure, whereas low-density stands showed the poorest performance. These findings suggest that both overly dense and sparse stands negatively affect spatial organization. Appropriate management practices, such as thinning or enrichment planting, are recommended to optimize stand structure and enhance ecological resilience.

## 1. Introduction

Stand structure can be categorized into non-spatial and spatial structures [1]. It reflects the fundamental functions and growth characteristics of a forest stand, playing a decisive role in its stability and sustainable development capacity, and serves as the theoretical foundation for forest management [2,3]. Analyzing the structural characteristics of plantation stands helps improve the quality of planting and management practices, while also enhancing understanding of the ecological status of these stands [4]. In recent years, structured forest management theory has evolved, emphasizing the maintenance of long-term stand stability and productivity through optimized spatial structure. This approach offers new perspectives and pathways for the sustainable management of planted forests [5].

Diameter distribution refers to the distribution of tree diameters across different size classes within a forest stand [6], playing a crucial role in stand growth, competition, and biomass [7]. Initial studies primarily explored diameter distribution patterns from a biological perspective. Subsequently, with the widespread adoption of mathematical expressions, probability density functions and growth equations became extensively applied to model stand diameter distribution. These include the Gamma distribution [8], Weibull distribution [9,10], negative exponential distribution [11], β distribution [12], normal distribution [13], as well as the logistic equation and Richard equation [14]. For instance, Li et al. [15] employed the Weibull and Gamma distributions to analyze and model the diameter distribution of natural forests in the Changhua River basin, thereby elucidating the distribution patterns of natural forest structure in this region.

Currently, research on forest spatial structure encompasses various forest types, including *Pinus tabulaeformis* [16], *Larix sibirica* [17], and *Pinus koraiensis* [18], with studies primarily focusing on analyzing stand spatial structure using different spatial structure parameters. Wang et al. [19] systematically analyzed the spatial structure characteristics of natural secondary larch forests in the Xing’an Mountains using backpack laser scanning (BLS) data. They calculated parameters such as angular scale, size ratio, and mixture degree from a multivariate distribution perspective. Li et al. [20] systematically analyzed seasonal rainforests in northern tropical karst regions by incorporating spatial error, spatial lag, and generalized additive models, revealing that stand spatial structure is a key factor regulating tree growth and seedling diversity. While these studies characterize specific aspects of stand spatial structure from diverse perspectives, they fail to accurately represent the holistic state of stand spatial structure. To overcome this limitation, Bi et al. [21] developed a new integrated evaluation system for stand spatial structure based on BLS data combined with the entropy weight method. They found that optimized thinning significantly enhances stand structural quality, further demonstrating that models from an integrated perspective can effectively evaluate and optimize stand spatial structure. This suggests that evaluating stand spatial structure from a comprehensive perspective facilitates more holistic control over the overall stand condition, providing more scientific decision-making support for structured forest management. In summary, to propose targeted scientific management measures for forests in climatic transition zones under climate change, it is necessary to establish a comprehensive evaluation index for the spatial structure of *Pinus taiwanensis* mixed forests and investigate their overall spatial structural state.

*Pinus taiwanensis* is a tree species endemic to China, playing a particularly vital role in mountain afforestation, reforestation, and ecological restoration in the country’s subtropical high-altitude regions [22]. It demonstrates significant ecological, social, and economic benefits across multiple domains [23]. In recent years, forestry scholars have conducted extensive research on the stand structure of *Pinus taiwanensis*. For instance, Bai et al. [24] analyzed the structural characteristics of *Pinus taiwanensis* plantations in Siming Mountains, Ningbo, with a particular focus on their spatial structure. Hou et al. [25] examined the diameter distribution of natural *Pinus taiwanensis* forests in Macheng, Hubei. Lü et al. [26] studied the impact of stand spatial structure on understory plant diversity in *Pinus taiwanensis* plantations at Huangbai Mountain Forest Farm in Dabie Mountains, Henan Province. However, there are few reports on the structural characteristics of *Pinus taiwanensis* plantations at different densities in climate transition zones [27,28].

This study investigates the diameter distribution of *Pinus taiwanensis* plantations in Huangbai Mountain Forest Farm, Xinyang City, Henan Province, using six probability distribution models. Four spatial structure parameters—full mixing degree, size ratio, angular scale, and forest layer index—were analyzed. The hierarchical analysis method uses the entropy weighting method to determine their weights, constructing four comprehensive indices of stand spatial structure. This investigation aims to explore the structural characteristics of the *Pinus taiwanensis* plantation at Huangbai Mountain Forest Farm in Xinyang City, Henan Province. Then provides a reference for structured forest management in this region and aids in formulating adaptive strategies for stand structure adjustment under climate change in transitional climatic zones, thereby promoting growth and regeneration for the stand.

Based on the research objectives, the following hypotheses were tested: H1: Among the six candidate diameter distribution models (Normal, Lognormal, Logistic, Gamma, Exponential, and Weibull), the Weibull and Gamma distributions are equally suitable for describing the diameter distribution across different densities. H2: Medium-density stands will exhibit a more optimal spatial structure than low- and high-density stands.

These hypotheses were tested using Kruskal–Wallis tests, Dunn’s post hoc tests with Bonferroni correction, bootstrap resampling (1000 iterations), and *AIC* comparisons, as detailed in the Statistical analysis section.

## 2. Results and Analysis

### 2.1. Diameter Distribution Characteristics of Pinus taiwanensis Plantations in the Climate Transition Zone

**Figure 1:**
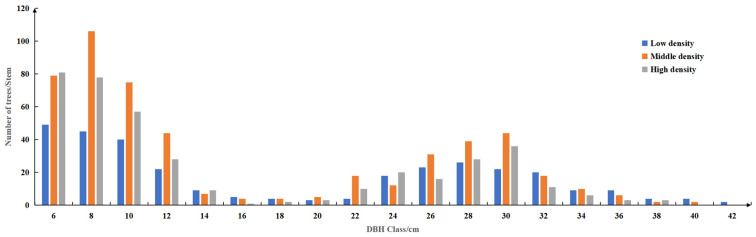
Diameter distribution under different forest stand densities.

According to the basic characteristics of the *Pinus taiwanensis* plantation sample plots (Table 1), the mean DBH of the plots was approximately 19.89 cm. As shown in Figure 1, trees in the 6–12 cm diameter class accounted for the highest proportion within the stands. With the continuous increase in diameter class, the variation in the number of trees differed among different stand densities. Specifically, low-density stands exhibited a pattern of initially decreasing, then increasing, and finally decreasing again; medium-density stands showed a trend of initially increasing, then decreasing, then increasing again, and finally decreasing; while high-density stands displayed a pattern of initially decreasing, then increasing, and then decreasing again. Overall, the number of trees in small diameter classes was relatively high across different stand densities. And as the stand density increases, the number of trees in large diameter classes in medium- and low-density stands was significantly higher than that in high-density stands, which is more favorable for stand regeneration. Therefore, for forest management in this region, appropriate tending and thinning measures should be implemented, particularly in high-density *Pinus taiwanensis* plantations, to adjust stand structure and promote tree growth.

Additionally, six different probability density function models were used to fit the diameter distribution of stands at varying densities (Table 2 and Table 3). The Weibull distribution model parameters for diameter distribution across different stand densities ranged from 0.88 to 1.16 and 17.67 to 26.71, respectively (Table 2). As shown in Table 3, the *p*-values for both the Gamma distribution and Weibull distribution models exceeded 0.05, indicating they passed the K-S test and are suitable for fitting the diameter distribution of *Pinus taiwanensis* plantations at different densities. As shown in Table 3, the *AIC* differences between the Gamma and Weibull models were less than 0.2 for all density classes (low: 148.29 vs. 148.44, Δ = 0.15; medium: 159.91 vs. 159.78, Δ = 0.13; high: 144.36 vs. 144.22, Δ = 0.14), which is well below the threshold of 2. Therefore, the Gamma and Weibull distributions are equally suitable for fitting the diameter distribution of *Pinus taiwanensis* plantations across different densities, with neither having a clear advantage over the other.

### 2.2. Spatial Distribution Characteristics of Pinus taiwanensis Plantations in the Climatic Transition Zone

**Figure 2:**
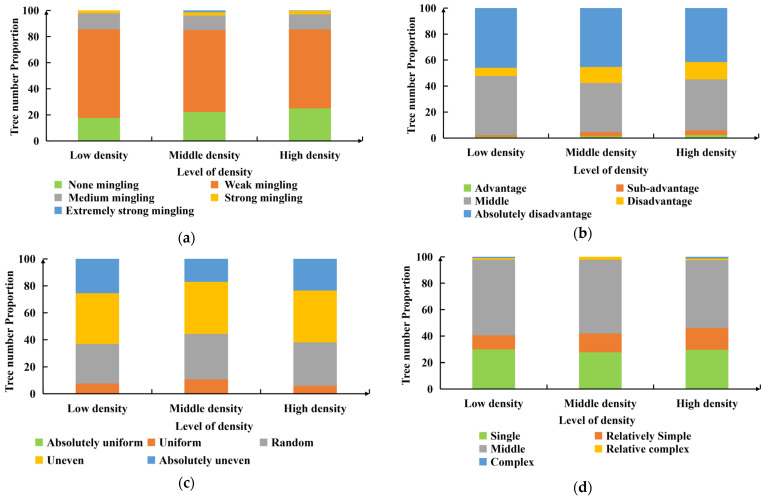
Frequency distribution of various spatial structure indicators under different stand densities ((**a**–**d**): full mixing degree, size ratio, angular scale, forest layer index).

The mean values and frequency distributions of spatial structure indices for *Pinus taiwanensis* plantations under different stand densities are presented in Table 4 and Figure 2. The degree of species segregation in stands of varying densities falls between zero and weak mixing. Their mean total mixing indices show little difference, with weak mixing accounting for a high proportion of trees (60.46–67.92%), while moderate mixing and above constitute only 14.47–15.02%. The proportion of zero-mixing trees was lowest in low-density stands at 17.61%, followed by medium-density stands, while high-density stands had the highest proportion at 25.00%. The proportion of extremely mixed trees was highest in medium-density stands at 1.38%, followed by high-density stands, while low-density stands had a proportion of 0. This indicates that both excessively low and high stand densities adversely affect species diversity. The degree of tree size differentiation and the proportion of each size class were relatively similar across different stand densities, with the average size ratio generally indicating a disadvantaged state. Moderate-sized trees accounted for the highest proportion (45.60%) in low-density stands, followed by high-density stands, while medium-density stands had the lowest proportion. Based on the standard interpretation of the uniform angle index, all stand densities exhibited a clumped (aggregated) spatial distribution, with average angular scales ranging from 0.659 to 0.725 (Table 4). Medium-density stands showed the highest proportion (44.07%) of trees distributed uniformly or randomly. The vertical structure complexity across different stand densities was relatively simple, with an average forest layer index ranging from 0.2251 to 0.2427.

### 2.3. Comprehensive Evaluation of Spatial Structure in Pinus taiwanensis Plantations in the Climatic Transition Zone

To rationally determine the importance of each parameter in the Analytic Hierarchy Process (AHP), and given that studies indicate strong correlations exist between Forest Structure Index (*FSI*) and these parameters, Pearson’s correlation coefficient was employed to analyze the relationships between *FSI* and each parameter. This analysis then established the relative importance of each parameter within the AHP framework. Table 5 indicates that *S* exhibits the strongest correlation with *FSI*, showing a highly negative relationship. The spatial structure parameters are ranked in descending order of magnitude as follows: forest layer index (*S*), full mixing degree (*Mc*), size ratio (*U*), and angular scale (*W*).

The comprehensive weights for each spatial structure parameter were determined using the Analytic Hierarchy Process (AHP) and entropy weight method, with the results shown in Table 6. *CI* and *CR* were used to determine whether the judgment matrix required adjustment. As shown in Table 6, *CR* = 0.00065 < 0.1, indicating no further adjustment was needed. Combining the entropy weight method, the composite weights for each parameter (*Mc*, *U*, *W*, *S*) were 0.2364, 0.2625, 0.1559, and 0.3451, respectively.

**Figure 3:**
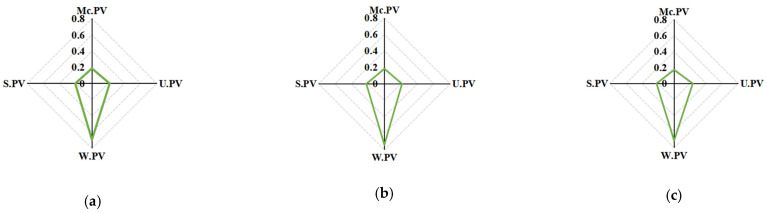
Radar diagram of the excellence coefficient of different spatial structure parameters under different densities ((**a**): low density; (**b**): medium density; (**c**): high density). Note: *Mc*.PV, *U*.PV, *W*.PV, and *S*.PV represent the average advantage coefficients for full mixing degree, size ratio, angular scale, and forest layer index, respectively.

Figure 3 displays the average excellence coefficients for spatial structure parameters across different densities. As shown, the average excellence coefficient for the angular scale is highest at 0.7405, while the average excellence coefficient for the full mixing degree is lowest at 0.1692. In the radar chart (Figure 3) depicting spatial structure parameters, larger shaded areas (*SPV*) indicate superior stand spatial structure. Therefore, as shown in Table 7, the ranking of stand spatial structure quality based on *SPV* across different densities is: medium density (0.1999) > high density (0.1991) > low density (0.1864).

The results of calculating the comprehensive index for evaluating different spatial structures under varying stand densities are shown in Table 7. As indicated in Table 7, the *Q* value is highest at low density (0.4445), followed by medium density, with the lowest value observed at high density. Medium density exhibited the highest *CDEV* and *CAPV* values at 0.2904 and 0.3508, respectively, followed by high density, while low density had the lowest *CDEV* and *CAPV* values. However, medium density recorded the lowest *FSI* value at 1.2300, high density was intermediate at 1.2347, and low density had the highest *FSI* value at 1.2480.

Kruskal–Wallis tests revealed that among the five indices, only *Q* showed a significant overall difference among the three density classes (χ^2^ = 7.554, *df* = 2, *p* = 0.023; Table 7). No significant differences were detected for *SPV* (*p* = 0.597), *FSI* (*p* = 0.619), *CDEV* (*p* = 0.597), or *CAPV* (*p* = 0.323).

Post hoc comparisons using Dunn’s test with Bonferroni correction were performed to identify which density classes differed significantly for the *Q* index. As shown in Table 8, the *Q* index differed significantly between low-density and high-density stands (*p =* 0.021), while no significant differences were found between low- and medium-density stands (*p* = 0.197) or between medium- and high-density stands (*p* = 1.000).

To assess the reliability of the mean estimates for the high-density class (*n* = 5), we performed bootstrap resampling with 1000 iterations for each spatial structure index. As shown in Supplementary Figure S1, the bootstrap distributions were narrow and centered around the original sample means. The 95% confidence intervals were relatively tight (e.g., *Q*: [0.305, 0.361]), and the coefficients of variation (*CV*) for all indices were below 0.14 (Supplementary Table S2). These results demonstrate that despite the small sample size, the mean estimates for the high-density class are stable and reliable.

Based on the comprehensive evaluation of five spatial structure indicators (*SPV*, *Q*, *FSI*, *CDEV*, *CAPV*), the spatial structure of *Pinus taiwanensis* plantations is optimal at medium density, followed by high density, with low density being the poorest. Therefore, spatial structure optimization and adjustment of low-density *Pinus taiwanensis* plantations is urgently needed.

## 3. Discussion

Stand structure is one of the key factors in evaluating forest ecological benefits. A reasonable stand structure is crucial for fully realizing the various functions of forest ecosystems. Due to their high planting density, artificial forests require a long period to naturally thin out, resulting in an unreasonable structure that adversely affects forest growth. Therefore, appropriate measures are needed to optimize the stand structure of plantation forests and promote healthy forest growth.

This study investigated the diameter distribution characteristics of *Pinus taiwanensis* plantations under varying stand densities. As a vital component of stand structure, diameter distribution reflects overall forest quality and growth development levels. The research results showed that as the diameter order increased, there were significant differences in the changes in tree numbers under different stand densities. As expected in typical stand structures, smaller diameter classes had higher tree numbers across all densities, while larger diameter classes were sparse in medium- and low-density stands. This phenomenon stems from low-density stands, where tree crowns gain ample nutritional space and reduced competition. Consequently, each tree receives greater access to nutrients, water, and sunlight, promoting growth and development. This finding aligns with Bilal Ahmad’s [29] research on North China larch plantation forests in Liupan Mountains, Ningxia: as stand density increases, individual tree volume decreases. Thus, reasonable thinning can adjust stand density to promote stand development. Consequently, plantation forest management practices must rationally regulate stand density to achieve high-quality tree growth and optimal stand structure adjustment.

Six probability density function models were applied to fit and compare the diameter distribution of stands under varying densities. Results indicate that both the gamma and Weibull distributions are suitable for describing the diameter distribution characteristics of *Pinus taiwanensis* plantations across different densities, with neither showing a clear advantage over the other (Δ*AIC* < 2 for all density classes).

Our Weibull parameter estimates (scale: 17.67–26.71, shape: 0.88–1.16) fall within the ranges typically reported for even-aged pine plantations. Saramäki [30] derived comparable Weibull parameters for *Pinus kesiya* plantations in Zambia. The consistency suggests that the diameter distribution patterns observed in our study are broadly representative of even-aged Pinus plantations.

The comparable performance of the Gamma and Weibull distributions in our study likely reflects the relatively narrow diameter range and the uniform, even-aged structure of the stands, where both distributions perform similarly well. This finding aligns with Cosenza et al. [31], who compared Johnson’s SB and Weibull functions for forest plantations and found that the Weibull performed comparably to the more flexible Johnson’s SB for *Pinus radiata* data, further supporting the robustness of the Weibull approach for pine plantation studies. The selection of appropriate diameter distribution models has important implications for forest management, as accurate diameter predictions are essential for yield estimation and silvicultural planning [30,32].

The spatial structure indices used in this study, while derived from static measurements, are closely linked to underlying biological processes. The mixing degree (*Mc*) reflects inter-specific competition and species coexistence, which develop over time through competitive exclusion or niche complementarity. The size ratio (*U*) captures size asymmetry and competitive hierarchy, which drives growth suppression of smaller trees and eventual mortality. The angular scale (*W*) indicates spatial resource utilization efficiency, with clumped patterns often resulting from patchy regeneration or micro-site heterogeneity. The forest layer index (*S*) describes vertical stratification, which influences light capture and photosynthetic efficiency over time. Thus, these indices serve as static proxies for dynamic processes that unfold over time.

Applying these indices to the study sites revealed the following spatial structure characteristics. Stand spatial structure influences stand stability and refinement management. Analysis of spatial structure characteristics in *Pinus taiwanensis* plantations at different densities revealed that although all plantations exhibited a clumped (aggregated) spatial distribution based on the uniform angle index (*W* > 0.517 for all density classes), with isolation levels between zero and weak mixed stands, and similar degrees of size stratification indicating a generally disadvantaged state, the vertical structure remained relatively simple. However, medium-density plantations and intensively mixed forests showed the highest proportion of dominant trees, with the greatest degree of species isolation. These stands exhibit minimal canopy shading and relatively open conditions, resulting in lower competition among trees—a pattern consistent with density-dependent competition dynamics observed in other plantation forests [30,32].

Different spatial structure parameters of forest stands are both interdependent and mutually influential. Relying on a single spatial structure parameter is insufficient to comprehensively describe the complex structure of a forest stand. To better understand and analyze forest stand structure, we must comprehensively consider multiple spatial structure parameters to accurately reveal the stand’s spatial characteristics, functions, and ecological benefits. Therefore, by utilizing a nonlinear weighting method to organically combine different spatial structure parameters and construct a comprehensive spatial structure evaluation index, we can effectively describe and reflect the spatial structure characteristics of forest stands while better identifying any irrationality in these spatial structure features. To optimize the assignment of weights to spatial structure parameters using the Analytic Hierarchy Process-Entropy Weighting Method and clarify the influence of each parameter on spatial structure, this study analyzed the correlation between the Spatial Structure Index (*FSI*) and individual parameters. This analysis established the following order of importance for parameters in pure plantation forests: Forest layer index (*S*) > Full mixing degree (*Mc*) > Size ratio (*U*) > Angular Scale (*W*). This approach enables a more rational configuration of the adjustment matrix, reduces subjective influences during weight assignment, and facilitates a more scientific and reasonable quantification of stand spatial structure status. Furthermore, the importance of forest layer index in this study has been corroborated by recent research. Sun [33] employed rough set theory to screen spatial structure indicators for four typical stands in Jindong Forest Farm, finding the forest layer index to be the most significant (0.124), followed by the competition index (0.096) and angular scale (0.084). In contrast, the simple mixing degree (0.043) and openness (0.043) exhibited lower importance, highlighting the critical role of the forest layer index in evaluating the spatial structure of plantation forests. Additionally, the spatial structure distance index *FSI*, average dominance coefficient index *SPV*, comprehensive distance evaluation index *CDEV*, comprehensive analysis method of indicator dominance coefficient *CAPV*, and comprehensive spatial structure index *Q* effectively assessed stand spatial structure quality. Their comprehensive evaluations of *Pinus taiwanensis* stands at different densities yielded consistent results: medium-density stands exhibited optimal spatial structure, followed by high-density stands, with low-density stands being the least favorable. For low-density *Pinus taiwanensis* plantations, their limited ecosystem functions stem from low biodiversity and vegetation cover. Therefore, reasonable dense planting in the later stage can be considered to increase biodiversity and vegetation coverage, and forest tending should be carried out based on scientific research and management experience to achieve long-term sustainable development.

It is worth noting that among the five comprehensive spatial structure indices evaluated in this study, the *Q* index (Single-tree Spatial Structure Comprehensive Index) showed optimal values in low-density stands (0.4445), while the other four indices (SPV, *FSI*, *CDEV,* and *CAPV*) indicated that medium-density stands exhibited the most optimal spatial structure. This discrepancy can be explained by the different structural attributes emphasized by each index. The *Q* index primarily reflects species mixing and neighborhood complexity, which can be enhanced in low-density stands due to greater spatial opportunities for species intermixing and reduced competitive exclusion [34]. In contrast, the other four indices incorporate additional structural dimensions: *SPV* emphasizes overall stand preference based on multiple spatial parameters, *FSI* focuses on stand stability, *CDEV* evaluates comprehensive distance-based spatial relationships, and *CAPV* assesses proximity patterns. These attributes tend to be optimized at moderate densities where competition is balanced but structure remains complex [30,32].

From a management perspective, this finding has important practical implications. If the primary management objective is to maximize species diversity and spatial complexity, low-density management (e.g., enrichment planting of companion species) may be considered. However, for most plantation forests where overall stability, productivity, and ecological resilience are the primary goals, medium-density management is recommended, as it optimizes the majority of spatial structure indices. This highlights the importance of multi-objective forest management planning, where density regulation should be tailored to specific management objectives. At the near-mature to mature stage (39–49 years), medium-density stands exhibited optimal spatial structure based on static measurements, which may indicate potential for higher stability and resilience, but long-term monitoring across different developmental stages is required to confirm this inference.

Several limitations of this study should be acknowledged. First, the sample plots were established using a purposive (typical) sampling method along the altitudinal gradient rather than through a strict random or systematic design. This approach ensures the inclusion of typical stand structures across the elevation gradient but may limit the generalization of the statistical results to the entire population of *Pinus taiwanensis* plantations in the region. Second, the high-density class contained only five plots (compared to nine plots in each of the other two classes). This imbalance reflects the natural rarity of high-density *Pinus taiwanensis* plantations in the study area due to limitations in light, nutrients, and historical management practices. To address this concern, we performed bootstrap resampling with 1000 iterations to assess the reliability of the mean estimates for the high-density class. The results showed narrow 95% confidence intervals and low coefficients of variation (*CV* < 0.14 for all five spatial structure indices; Supplementary Table S2, Supplementary Figure S1), indicating that the mean estimates are stable despite the small sample size. Nevertheless, future studies with larger sample sizes of high-density stands are warranted to validate our findings. Third, larger datasets are needed to analyze stand structural characteristics more accurately. Fourth, we have not yet considered the impact of trees smaller than 5 cm on stand structure, which warrants further investigation. Fifth, as a static (single-time) study, we acknowledge that our measurements represent snapshots rather than dynamic trajectories. Future research should establish permanent plots with repeated measurements to track changes in maximum diameter, maximum density, and the temporal evolution of spatial structure indices across different developmental stages (e.g., young, middle-aged, near-mature, and mature stands). Such dynamic data would enable estimation of marginal productivity and provide direct evidence for the biological processes underlying the observed patterns.

Recent rapid advancements in UAV LiDAR and deep learning technologies offer new possibilities for overcoming the limitations of traditional survey methods. The Tree-Net model proposed by Jarahizadeh and Salehi [34], optimized based on the YOLO deep learning framework, achieved a 34% improvement in F1-score for individual tree detection compared to traditional YOLO on a dataset covering approximately 25,000 coniferous and broadleaf trees. It also demonstrated a 60% increase in training efficiency, providing a powerful tool for large-scale, high-efficiency forest surveys using UAV LiDAR data. The introduction of such advanced technologies holds promise for achieving precise identification and structural parameter extraction of trees across different diameter classes—including saplings under 5 cm in diameter. This significantly enhances the accuracy and efficiency of forest stand structure surveys, enabling more accurate evaluations of forest structural characteristics. Such advancements provide a theoretical foundation for the scientific management of *Pinus taiwanensis* plantations in this region.

## 4. Materials and Methods

### 4.1. Study Area and Data

The study area is located in the Jiufengjian Forest District of Huangbai Mountain Forest Farm, Shangcheng County, Xinyang City, Henan Province (115°16′52″–115°23′5″ E, 31°22′44″–31°30′2″ N) within the Dabie Mountains. This area straddles the boundary between the northern subtropical and warm temperate climate zones. Following the Köppen–Geiger climate classification system [35,36], this region falls within the transition between Cfa (humid subtropical) and Cwa (monsoon-influenced humid subtropical) zones. Quantitatively, it is characterized by an annual mean temperature of 15.4 °C and an annual precipitation of 1509.0 mm, with both values falling near the median of the two adjacent climate zones. The region features distinct seasons, ample rainfall, and a mean relative humidity of 76%. The soil is subtropical yellow-brown soil, which is rich in organic matter, has strong water and nutrient retention capacity, and is mildly acidic. The study area supports abundant plant resources, with *Pinus taiwanensis*, *Cunninghamia lanceolata*, *Diospyros lotus*, *Platycarya strobilacea*, and *Malus spectabilis* as the primary tree species.

**Figure 4:**
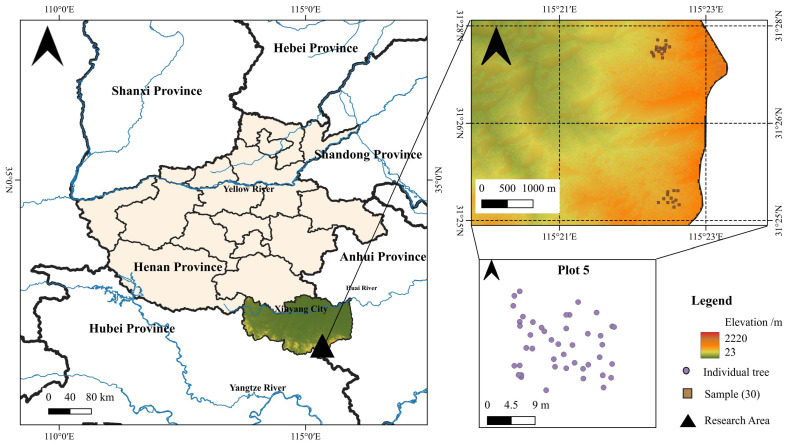
Sample plot distribution map.

In 2020, a total of 30 rectangular plots of 20 m × 30 m were established along an altitudinal gradient using a typical sampling method (a form of purposive sampling) in Huangbai Forest Farm, Shangcheng County, Xinyang City, Henan Province (Figure 4). The plots were from *Pinus taiwanensis* plantations aged 39–49 years, classified as near-mature to mature stands. This approach involves the deliberate placement of plots to capture the representative range of site conditions [37] (e.g., altitude, slope aspect, soil type) where *Pinus taiwanensis* plantations occur, rather than following a strict random or systematic design. A full callipering survey was conducted from July to August 2020 for all living trees with diameter at breast height (DBH) ≥ 5 cm within the plots, following the standard forest inventory threshold for the tree layer in temperate and subtropical forests of China [38]. Taking the southwest corner of each plot as the coordinate origin, basic information for each tree, including tree number, species name, tree coordinates within the plot, DBH, tree height, clear bole height, and crown width, was measured and recorded. Simultaneously, basic conditions for each plot, such as altitude, slope gradient, slope aspect, slope position, age group, and canopy density, were recorded. This purposive sampling may limit the generalizability of statistical inferences, but it ensures the inclusion of typical stand structures across the elevation gradient.

Based on the basal area composition ratio of each tree species, plots where the basal area proportion of *Pinus taiwanensis* was less than 65% were excluded, resulting in a total of 23 *Pinus taiwanensis* plantation plots. Considering site conditions such as topographic factors and soil types, the stand density of the 23 plots was classified into three different density classes: low-density stands (L) (<700 trees·ha^−1^), totaling 9 plots; medium-density stands (M) (700 trees ha^−1^ ≤ density < 1000 trees ha^−1^), totaling 9 plots; and high-density stands (H) (≥1000 trees·ha^−1^), totaling 5 plots. This sample size (23 plots total) is comparable to similar studies on plantation stand structure [39,40]. The unbalanced classification reflects the natural distribution of stand densities in the study area, where high-density *Pinus taiwanensis* plantations are inherently less common due to limitations in light, nutrients, and historical management practices. The 5 plots in the high-density class represent the entire available population meeting our selection criteria within the sampled altitudinal gradient. The stand characteristics of each plot are presented in Table 1.

### 4.2. Diameter Distribution Fitting

In this study, six probability density distributions—one single-parameter (Exponential distribution) and five two-parameter (Normal distribution, Lognormal distribution, Logistic distribution, Gamma distribution, and Weibull distribution) (Table 9)—were used to fit the stand diameter distribution of *Pinus taiwanensis* plantations under different densities. The Exponential distribution, a special case of the Gamma and Weibull (shape = 1), was retained as a nested model for comparison. On this basis, the six distribution models were compared and analyzed to select the optimal stand diameter structure distribution model. The Kolmogorov–Smirnov (K-S) test was employed, and two information criteria, *AIC* (Equation (1)) and *BIC* (Equation (2)), were used to compare and screen the optimal fitting functions. The Kolmogorov–Smirnov (K-S) test is a method for testing whether a single data sample conforms to a specific distribution form. When the *p*-value is greater than 0.05, it indicates that the data sample follows that specific distribution, meaning the model is accepted [27]; conversely, if the *p*-value is less than or equal to 0.05, the model is rejected. Furthermore, *BIC* is more stringent than *AIC*.

(1) $$AIC=2p+n\ln\left(\frac{{SSE}_{P}}{n}\right)$$

(2) $$BIC=p\left(\ln n\right)+n\ln\left(\frac{{SSE}_{P}}{n}\right)$$

where *p* represents the number of parameters in the distribution model; *n* represents the number of observations; SSE_p_ represents the residual sum of squares. Smaller *AIC* and *BIC* values indicate a better fitting effect. When the difference in *AIC* (Δ*AIC*) between two models is less than 2, both models have substantial empirical support and can be considered equally plausible [41].

### 4.3. Calculation of Spatial Structure Indices

#### 4.3.1. Construction of Spatial Structure Units and Edge Correction

To more accurately analyze the structural characteristics of *Pinus taiwanensis* plantations under different stand densities in the transition zone, this study comprehensively analyzed existing research results and combined them with the theory of structure-based forest management. The method of using the four nearest neighboring trees was adopted to construct spatial structure units for analyzing the spatial structure characteristics of *Pinus taiwanensis* plantations. However, during the division of stand spatial structure units, the tree spatial structure units constructed with boundary trees at the plot edges as the center trees may have the problem of incompleteness, as their neighboring trees might be located outside the plot. This is detrimental to accurately quantifying the stand spatial structure. This study employed the eight-neighbor translation method for edge correction. Specifically, the original plot was replicated in eight directions, i.e., up, down, left, right, top-left, bottom-left, top-right, and bottom-right, forming a large plot containing nine sub-plots to eliminate edge effects [42]. The trees in the extended plots were only considered as neighbors to the boundary trees of the original plot, and their spatial structure parameters were not calculated when they served as center trees. This ensures that trees at the edges are also fully considered, making the analysis of stand spatial structure more accurate.

#### 4.3.2. Selection of Spatial Structure Parameters

To analyze the spatial structure characteristics of *Pinus taiwanensis* plantations with different densities in Huangbai Forest Farm, Xinyang City, Henan Province, this study selected the full mixing degree (*Mc*) [43] to characterize the degree of spatial segregation of trees, tree species diversity, and the uniformity of each species’ proportion; the size ratio (*U*) [44] to depict the degree of size differentiation among trees; the angular scale (*W*) [45] to describe the horizontal spatial distribution pattern of trees; and the forest layer index (*S*) [46] to reflect the diversity of forest layers. The calculation formulas for the four spatial structure parameters (Table 10) and the meanings represented by their different values (Table 11) are summarized as follows:

### 4.4. Construction of Stand Spatial Structure Comprehensive Index

Based on the structural characteristics of *Pinus taiwanensis* plantations, this study adopted a combined subjective-objective weighting method integrating the analytic hierarchy process (AHP) and the entropy weight method to determine and assign weights to four spatial structure parameters: full mixing degree, angular scale, size ratio, and storey index. In the AHP, a judgment matrix was constructed using each spatial structure parameter as an indicator. The consistency ratio *CR* (*CR* = *CI*/*RI*, where *CI* is the consistency index and RI is the random consistency index) was used to assess whether the judgment matrix was acceptable. If acceptable, the weight vector was taken as the subjective weight (*v_i_*) for each indicator; otherwise, the judgment matrix was adjusted. The entropy weight method was used to determine the objective weight (*w_i_*) (Equation (3)). Due to the presence of zero values among the indicators composed of the structural parameters, data standardization of the parameter values was required (Equation (4)). A lower entropy value indicates more information reflected by the indicator. Finally, the subjective and objective weights were combined to obtain the comprehensive weight ${CW}_{i}$ (Equation (5)), which was used to construct the stand spatial structure comprehensive evaluation index.

(3) $$w_{i}=\frac{1-E_{i}}{m-\sum_{i=1}^{m}E_{i}}\begin{array}{cc} , & E_{i}=-\sum_{j=1}^{n}P_{ij}\ln P_{ij} \end{array}$$

where $w_{i}$ is the objective weight of the i-th structural parameter; $E_{i}$ is the information entropy of the *i*-th structural parameter; $P_{j}$ is the proportion of the *j*-th observation value of the *i*-th structural parameter relative to that parameter; *m* is the number of structural parameters; *n* is the number of samples; $P_{ij}=\frac{X_{ij}^{\prime }}{\sum_{j=1}^{n}X_{ij}^{\prime }}$, where $X_{ij}^{\prime }$ is the dimensionless value of the *j*-th observation value of the *i*-th structural parameter.

(4) $$X_{ij}^{\prime }=\left\{\begin{array}{c} \frac{X_{ij}-min\left(X_{ij}\right)}{\max\left(X_{ij}\right)-min\left(X_{ij}\right)}\begin{array}{cc} , & if\;X_{ij}\;is\;a\;positive\;indicator, \end{array}\\ \frac{max\left(X_{ij}\right)-X_{ij}}{\max\left(X_{ij}\right)-min\left(X_{ij}\right)}\begin{array}{cc} , & \mathrm{if}\;X_{ij}\;is\;a\;negative\;indicator, \end{array}\\ \frac{max\left(\left|X_{ij}-k\right|\right)-\left|X_{ij}-k\right|}{\max\left(\left|X_{ij}-k\right|\right)-min\left(\left|X_{ij}-k\right|\right)}\begin{array}{cc} , & \mathrm{if}\;X_{ij}\;is\;a\;moderate\;indicator. \end{array} \end{array}\right.$$

where $X_{ij}$ is the actual value of the *j*-th observation of the *i-th* structural parameter, and k is the moderate value of the moderate indicator, taking a value of 0.5. Among these, the positive indicators include the full mixing degree and the forest layer index, the negative indicator is the size ratio, and the moderate indicator is the angular scale.

(5) $${CW}_{i}=\frac{w_{i}v_{i}}{\sum_{i=1}^{m}w_{i}v_{i}}$$

where ${CW}_{i}$ is the comprehensive weight of the *i*-th structural parameter; $w_{i}$ is the objective weight of the *i*-th structural parameter; $v_{i}$ is the subjective weight of the *i*-th structural parameter.

Bu [47] proposed using preference values to characterize the gap between the actual state and the ideal state of a forest, thereby representing the internal spatial structure of the forest. In this context, the angular scale is a moderate indicator with an optimal value (*O*) of 0.5; the full mixing degree and forest layer index are positive indicators with optimal values (*O*) of 1; and the size ratio is a negative indicator with an optimal value (*O*) of 0. Therefore, the calculation formula for the average superiority degree (*PV_i_*) of each spatial structure parameter is shown in Equation (6). An individual tree spatial structure comprehensive index (*Q_i_*) can be constructed to comprehensively evaluate the spatial structure characteristics of the stand.

(6) $${PV}_{i}=1-\sqrt{\frac{\sum_{j=1}^{n}{\left(I_{j}-O_{i}\right)}^{2}}{n}}$$

where $I_{j}$ is the actual value of the space structure parameter *i* of the *j*-th space structure unit; $O_{i}$ is the optimal value of the *i*-th spatial structure parameter.

Due to the certain correlations among the four spatial structure parameters, the nonlinear weighting method [48] was used to optimize the stand spatial structure distance index *FSI* [49], the comprehensive distance evaluation index *CDEV* [50], the comprehensive analysis method of indicator superiority coefficient *CAPV* [51], and the spatial structure comprehensive index *Q* [52] (see Table 12). Combined with the average superiority coefficient index *SPV* [53], the spatial structure characteristics were comprehensively evaluated. Among these, a smaller *FSI* value indicates a better stand spatial structure; larger values of *CDEV*, *CAPV*, *Q*, and *SPV* indicate a closer proximity to the ideal spatial structure.

### 4.5. Statistical Analysis

All statistical analyses were conducted in R version 4.2.0 [54]. Given the unbalanced design (low-density: *n* = 9, medium-density: *n* = 9, high-density: *n* = 5) and the small sample size of the high-density class, non-parametric tests were employed for comparisons among the three density classes. Specifically, the Kruskal–Wallis test was used to detect overall differences, followed by Dunn’s test with Bonferroni correction for pairwise comparisons. These tests do not assume normality or equal variances, making them appropriate for our data.

To evaluate the reliability of mean estimates for the spatial structure indices in the high-density class (*n* = 5), we performed bootstrap resampling with 1000 iterations to generate 95% confidence intervals [55]. The coefficient of variation (*CV*) was also calculated as an additional measure of within-group stability.

## 5. Conclusions

Using *Pinus taiwanensis* plantations in climatic transition zones as the study subject, six probability density distribution models were employed to fit the diameter distribution patterns of stands at different densities. Four spatial structure parameters were utilized to construct five comprehensive spatial structure evaluation indices through the Analytic Hierarchy Process-Entropy Weighting method combined with the average excellence coefficient. These indices comprehensively evaluated the spatial structure characteristics of stands at varying densities. The findings ultimately concluded that medium-density *Pinus taiwanensis* plantations exhibit optimal spatial structure. High-density stands exhibit intermediate spatial structure, requiring subsequent appropriate tending and regeneration. Low-density stands demonstrate the poorest spatial structure, necessitating underplanting to establish mixed forests and optimize stand structure.

## Figures and Tables

**Table 1:** Stand characteristic factors for each sample plot.

Density Class	Plot No.	Altitude (m)	Canopy Density	Mean DBH (cm)	Mean Tree Height (m)	Number of Trees per Hectare (Trees·ha^−1^)	Basal Area per Hectare (m^2^·ha^−1^)	Stand Age (Year)	Age Class
Low density (L)	2	778.3	0.82	18.3	10.1	595	15.66	39	Near-mature
	3	790.1	0.76	19.9	10.5	522	16.19	39	Near-mature
	4	756.2	0.82	26.6	12.1	265	14.73	39	Near-mature
	5	813.5	0.78	20.2	10.7	511	16.41	39	Near-mature
	6	823.2	0.83	20.4	10.9	419	13.69	39	Near-mature
	8	834.5	0.78	22.7	11.5	440	17.77	49	Mature
	14	939.8	0.7	18.6	9.9	558	15.13	49	Mature
	26	840.9	0.86	24.6	12.8	218	10.35	39	Near-mature
	27	840.9	0.83	25.3	13.0	430	21.6	39	Near-mature
Medium density (M)	12	853.8	0.75	18.0	9.8	899	22.83	49	Mature
	15	944.3	0.72	18.6	10.0	737	20.03	49	Mature
	17	912.7	0.76	16.2	10.8	881	18.22	49	Mature
	18	909.2	0.8	19.6	11.5	714	21.45	49	Mature
	19	839	0.75	20.1	11.2	873	27.57	39	Near-mature
	22	829.4	0.86	16.8	9.7	858	19.01	39	Near-mature
	24	824	0.9	18.9	10.6	788	22.08	39	Near-mature
	28	792.7	0.82	20.7	12.2	815	27.38	39	Near-mature
	29	794.2	0.78	21.8	13.2	975	36.54	39	Near-mature
High density (H)	1	839	0.76	18.9	10.3	1081	30.22	39	Near-mature
	11	886.9	0.74	17.2	9.3	1152	26.69	49	Mature
	21	820.1	0.85	17.9	10.4	1005	25.41	39	Near-mature
	23	862.2	0.8	16.5	10.1	1196	25.63	39	Near-mature
	30	782.4	0.83	20.0	10.9	1063	33.45	39	Near-mature

**Table 2:** Fitting parameters of different DBH distribution models for different forest stand densities.

Distributional Models	Parameters	Density Level		
		Low Density (L)	Medium Density (M)	High Density (H)
Normal	*μ*	16.74 ± 3.31	28.11 ± 7.09	23.06 ± 6.1
	*σ*	14.41 ± 2.34	30.07 ± 5.01	25.14 ± 4.31
Lognormal	μ	2.39 ± 0.22	2.67 ± 0.29	2.44 ± 0.31
	σ	0.98 ± 0.16	1.23 ± 0.21	1.29 ± 0.22
Logistic	a	14.9 ± 3.3	23.14 ± 6.69	18.56 ± 5.59
	b	8.18 ± 1.56	16.32 ± 3.28	13.39 ± 2.81
Gamma	α	1.31 ± 0.38	0.88 ± 0.26	0.84 ± 0.25
	β	0.08 ± 0.03	0.03 ± 0.01	0.04 ± 0.01
Exponential	λ	0.06 ± 0.01	0.04 ± 0.01	0.04 ± 0.01
Weibull	a	1.16 ± 0.21	0.9 ± 0.17	0.88 ± 0.17
	b	17.67 ± 3.7	26.71 ± 7.38	21.6 ± 6.28

Note: The values in each column are estimated parameters ± standard error.

**Table 3:** Parameter test of different DBH distribution models with different stand densities.

Distributional Models	Indicators	Density Level		
		Low Density (L)	Medium Density (M)	High Density (H)
Normal	D	0.98	0.98	0.92
	*p*	0.00	0.00	0.00
	*AIC*	159.30	177.61	161.88
	*BIC*	161.19	179.39	163.54
Lognormal	D	0.81	0.81	0.75
	*p*	0.00	0.00	0.00
	*AIC*	147.83	158.84	143.88
	*BIC*	149.72	160.63	145.54
logistic	D	0.90	0.88	0.83
	*p*	0.00	0.00	0.00
	*AIC*	159.61	176.99	161.06
	*BIC*	161.49	178.78	162.73
Gamma	D	0.17	0.14	0.14
	*p*	0.66	0.87	0.90
	*AIC*	148.29	159.91	144.36
	*BIC*	150.17	161.69	146.02
Exponential	D	0.90	0.87	0.83
	*p*	0.00	0.00	0.00
	*AIC*	147.07	158.10	142.69
	*BIC*	148.01	158.99	143.53
Weibull	D	0.16	0.13	0.13
	*p*	0.70	0.92	0.92
	*AIC*	148.44	159.78	144.22
	*BIC*	150.33	161.56	145.89

**Table 4:** Mean values of various spatial structure indicators under different forest stand densities.

Density Level	Structural Indicators			
	Full Mixing Degree (Mc)	Size Ratio (U)	Angular Scale (W)	Forest Layer Index (S)
Low density (L)	0.1930	0.7398	0.7250	0.2251
Medium density (M)	0.1955	0.7424	0.6589	0.2386
High density (H)	0.1819	0.7192	0.7097	0.2427

**Table 5:** Correlation Test Results Between *FSI* Mean Values and Mean Values of Spatial Structural Parameters.

Spatial Structure Parameters	Full Mixing Degree (*Mc*)	Size Ratio (*U*)	Angular Scale (*W*)	Forest Layer Index (*S*)
Correlation coefficient	−0.6300	0.3136	0.2386	−0.8144
*p* value	0.0013	0.1451	0.2730	0.0000

**Table 6:** Consistency Testing and Weights.

Name	Spatial Structure Parameters			
	Full Mixing Degree (*Mc*)	Size Ratio (*U*)	Angular Scale (*W*)	Forest Layer Index (*S*)
Full mixing degree (*Mc*)	1	3/2	3/2	2/3
Size ratio (*U*)	2/3	1	1	1/2
Angular scale (*W*)	2/3	1	1	1/2
Forest layer index (*S*)	3/2	2	2	1
Consistency check	*CI* = 0.00058, *CR* = 0.00065			
Subjective weight	0.2641	0.1813	0.1813	0.3734
objective weight	0.2169	0.3508	0.2084	0.2239
Integrated weights	0.2364	0.2625	0.1559	0.3451

**Table 7:** Comprehensive evaluation results of stand spatial structure under different stand densities (mean ± SE).

Density Level	Number of Plots	Comprehensive Evaluation Indicators for Spatial Structure				
		*SPV*	*Q*	*FSI*	*CDEV*	*CAPV*
Low density (L)	9	0.1864 ± 0.0122	0.4445 ± 0.0256	1.2480 ± 0.0169	0.2803 ± 0.0090	0.3339 ± 0.0107
Medium density (M)	5	0.1999 ± 0.0083	0.3707 ± 0.0275	1.2300 ± 0.0083	0.2904 ± 0.0054	0.3508 ± 0.0094
High density (H)	5	0.1991 ± 0.0103	0.3328 ± 0.0120	1.2347 ± 0.0163	0.2877 ± 0.0082	0.3442 ± 0.0111
*p*-value (Kruskal–Wallis)		0.597	0.023 *	0.619	0.597	0.323

Note: *SPV*, Stand Structure Preference Value; *Q*, Single-tree Spatial Structure Comprehensive Index; *FSI*, Forest Stability Index; *CDEV*, Comprehensive Distance Evaluation; *CAPV*, Comprehensive Assessment of Proximity Vector. Kruskal–Wallis test was used to compare indices among the three density classes. * *p* < 0.05 indicates significant overall difference among density classes. Post hoc Dunn’s test with Bonferroni correction revealed that *Q* differed significantly between low- and high-density stands (*p* = 0.021).

**Table 8:** Results of Dunn’s post hoc test with Bonferroni correction for the Single-tree Spatial Structure Comprehensive Index (*Q*).

Comparison	*n*_1_	*n*_2_	Statistic	*p*-Value	*p*-Adjusted	Significance
Low density (L) vs. Medium density (M)	9	9	1.84	0.0657	0.197	ns
Low density (L) vs. High density (H)	9	5	2.7	0.0069	0.021	*
Medium density (M) vs. High density (H)	9	5	0.93	0.3525	1	ns

Note: *n*_1_ and *n*_2_ indicate the number of plots in each compared group (*n* = 9 for L and M, *n* = 5 for H); ns, not significant. Dunn’s test with Bonferroni correction was used for pairwise comparisons following a significant Kruskal–Wallis test. * *p* < 0.05 indicates a statistically significant difference between the two density classes.

**Table 9:** Density functions of six continuous probability distributions.

Distribution Name	Probability Density Function	Number of Parameters
Normal distribution	$f\left(x\right)=\frac{1}{\sqrt{2\pi }\sigma x}e^{\frac{{\left(x-\mu \right)}^{2}}{2\sigma^{2}}}$	2
Lognormal distribution	$f\left(x\right)=\frac{1}{\sqrt{2\pi }\sigma x}e^{\frac{{\left(\ln\left(x\right)-\mu \right)}^{2}}{2\sigma^{2}}}$	2
Logistic distribution	$f\left(x\right)=\frac{1}{1+e^{\frac{-\left(x-a\right)}{b}}}$	2
Gamma distribution	$f\left(x\right)=\frac{1}{\Gamma \left(x\right)\beta^{\alpha }}x^{\alpha -1}e^{\frac{x}{\beta }}$	2
Exponential distribution	$f\left(x\right)=\lambda e^{-\lambda x}$	1
Weibull distribution	$f\left(x\right)=\frac{k}{l}{\left(\frac{x}{l}\right)}^{k-1}e^{-{\left(\frac{x}{l}\right)}^{k}}$	2

Note: *μ* and *a* are location parameters; *σ*, *b*, *β* and *λ* are scale parameters; *α* and *k* are shape parameters; *l* is a scale parameter. The Exponential distribution is a one-parameter distribution and is a special case of the Gamma and Weibull distributions (shape parameter = 1). It is included as a nested model for comparative purposes.

**Table 10:** Stand spatial structure parameters.

Parameters	Formula
Full mixing degree (Mc)	${Mc}_{i}=\frac{1}{2}\left(D_{i}+\frac{c_{i}}{n_{i}}\right)\times M_{i}$ $\begin{array}{cc} where,\;\; & \left\{\begin{array}{c} M_{i}=\frac{1}{n_{i}}\sum_{j=1}^{n_{i}}v_{ij}\begin{array}{cc} , & \left\{\begin{array}{c} If\;subject\;tree\;i\;and\;neighbor\;j\;are\;different\;species,v_{ij}=1\\ otherwise,\;v_{ij}=0 \end{array}\right. \end{array}\\ D_{i}=1-\sum_{k=1}^{s_{ik}}p_{ik}^{2} \end{array}\right. \end{array}$
Size ratio (U)	$U_{i}=\frac{1}{n_{i}}\sum_{j=1}^{n_{i}}k_{ij}\begin{array}{cc} , & \left\{\begin{array}{c} \mathrm{If}\;\mathrm{the}\;\mathrm{DBH}\;\mathrm{of}\;\mathrm{neighbor}\;j\;\mathrm{is}\;\mathrm{greater}\;\mathrm{than}\;\mathrm{that}\;\mathrm{of}\;\mathrm{subject}\;\mathrm{tree}\;i\;,\;\;k_{ij}=1\\ otherwise,\;k_{ij}=0 \end{array}\right. \end{array}$
Angular scale (W)	$W_{i}=\frac{1}{n_{i}}\sum_{j=1}^{n_{i}}z_{ij}\begin{array}{cc} , & \left\{\begin{array}{c} \;If\;the\;angle\;between\;the\mathrm{subject}\;\mathrm{tree}\;i\;and\;the\mathrm{neighbor}\;j\;is\;less\;than\;the\;standard\;angle,\;z_{ij}=1\\ otherwise,\;z_{ij}=0 \end{array}\right. \end{array}$
Forest layer index (S)	$S_{i}=\frac{Z_{i}}{3}\times \frac{1}{n_{i}}\sum_{j=1}^{n_{i}}s_{ij}\begin{array}{cc} , & \left\{\begin{array}{c} If\;the\;subject\;tree\;i\;and\;neighbor\;j\;belong\;to\;different\;storeys,\;s_{ij}=1\\ otherwise,\;s_{ij}=0 \end{array}\right. \end{array}$

Note:${\;Mc}_{i}$, $U_{i}$, $W_{i}$, $S_{i}$ are the full mixing degree, size ratio, angular scale, and forest layer index of the subject tree in the *i*-th spatial structure unit, respectively; $n_{i}$, $c_{i}$ are the number of nearest neighboring trees around the subject tree in the *i*-th spatial structure unit and the number of adjacent neighbor pairs that are of different species among the nearest neighboring trees, respectively; $D_{i}$ is the Simpson index for the *i*-th spatial structure unit; $M_{i}$ is the simple mixing degree; $p_{ik}$ and $s_{ik}$ are the proportion of individuals and the number of tree species for the *k*-th tree species in the *i*-th spatial structure unit; $v_{ij},\;\;k_{ij}$, $z_{ij}$ and $s_{ij}$ are discrete variables: *i* refers to the subject tree; *j* refers to the neighboring tree.

**Table 11:** Value meanings of different spatial structure parameters.

Spatial Structure Parameter	Value	Meaning
Full mixing degree (*Mc*)	0	Zero mixing
	(0, 0.25]	Weak mixing
	(0.25, 0.50]	Moderate mixing
	(0.50, 0.75]	Strong mixing
	(0.75, 1.00]	Very strong mixing
Size ratio (*U*)	0	Dominant
	0.25	Sub-dominant
	0.5	Intermediate
	0.75	Inferior
	1	Absolute inferior
Angular scale (*W*)	0	Absolutely uniform
	0.25	Uniform
	0.5	Random
	0.75	Clumped
	1	Absolutely clumped
Forest layer index (*S*)	0	Single-layered
	(0, 0.25]	Relatively simple
	(0.25, 0.50]	Moderate
	(0.50, 0.75]	Relatively complex
	(0.75, 1.00]	Complex

**Table 12:** Comprehensive evaluation indices of spatial structure.

Evaluation Index	Formula
*Q*	$Q=\sum_{i=1}^{m}\left({CW}_{i}\times X_{i}^{\prime }\right)$
*FSI*	$FSI=\sqrt{{\left(\bar{M}-1\right)}^{2}+{\left(\bar{U}-0.25\right)}^{2}+{\left(\bar{W}-0.5\right)}^{2}+{\left(\bar{S}-1\right)}^{2}}$
*CDEV*	$CDEV=\sum_{i=1}^{m}\left({CW}_{i}\times {PV}_{i}\right)$
*CAPV*	$CAPV=\sqrt{\sum_{i=1}^{m}\left({CW}_{i}\times {PV}_{i}^{2}\right)}$

Note: ${CW}_{i}$ and ${PV}_{i}$ are the comprehensive weight and average dominance of the *i*-th spatial structure parameter; *m* is the number of spatial structure parameters; $\bar{M}$,$\;\bar{U}$, $\bar{W}$ and $\bar{S}$ are the mean values of each spatial structure unit *M*, *U*, *W* and *S*.

## Data Availability

The data presented in this study are not publicly available due to restrictions related to data confidentiality.

## Supplementary Materials

The following supporting information can be downloaded at: https://www.mdpi.com/article/10.3390/plants15121842/s1, Table S1: Descriptive statistics of spatial structure indices by density class; Table S2: Bootstrap 95% confidence intervals for spatial structure indices in the high-density class (n = 5, 1000 iterations); Figure S1: Bootstrap 95% confidence intervals for spatial structure indices in the high-density class (n = 5) (Blue dashed line: original mean. Green dotted lines: 95% confidence interval. Upper-right corner: mean, 95% CI, and coefficient of variation (CV). Narrow distributions and low CVs (all < 0.14) indicate reliable mean estimates despite the small sample size). Note: SPV, Q, FSI, CDEV and CAPV are as defined in Table 11.
