# Supplementary material for: Structural Characteristics Analysis of Pinus taiwanensis Plantation in Climate Transition Zone
Source: Plants (Basel). 2026 Jun 14;15(12):1842. doi: 10.3390/plants15121842 (PMC13306897; doi:10.3390/plants15121842)
Supplement: Supplementary file 1 [file plants-15-01842-s001.zip › plants-4283625-supplementary.pdf]

Supplementary Table S1. Descriptive statistics of spatial structure indices by density class.

Density class	*n*	Statistical index	Comprehensive evaluation indicators for spatial structure				
			SPV	Q	FSI	CDEV	CAPV
L	9	Mean	0.1898	0.4445	1.248	0.2803	0.3339
		SD	0.0366	0.0769	0.0506	0.027	0.0321
		SE	0.0122	0.0256	0.0169	0.009	0.0107
		Median	0.1971	0.4287	1.2503	0.2871	0.3385
		Min	0.1477	0.3303	1.1651	0.2421	0.2922
		Max	0.2548	0.5686	1.3482	0.327	0.3866
M	9	Mean	0.1972	0.3707	1.23	0.2904	0.3508
		SD	0.0248	0.0824	0.0249	0.0163	0.0283
		SE	0.0083	0.0275	0.0083	0.0054	0.0094
		Median	0.196	0.3623	1.2328	0.288	0.3602
		Min	0.1689	0.2831	1.1994	0.2693	0.2973
		Max	0.2389	0.5539	1.2547	0.3164	0.3798
H	5	Mean	0.1984	0.3328	1.2347	0.2877	0.3442
		SD	0.023	0.0268	0.0364	0.0183	0.0249
		SE	0.0103	0.012	0.0163	0.0082	0.0111
		Median	0.2133	0.3235	1.2284	0.2959	0.3511
		Min	0.1607	0.3003	1.1615	0.2602	0.3005
		Max	0.2246	0.3967	1.2805	0.3155	0.3685

Note: L, low-density; M, medium-density; H, high-density; SD, standard deviation; SE, standard error; n, number of plots. SPV, Q, FSI, CDEV and CAPV are as defined in Table 11.

Supplementary Table S2. Supplementary Table S2. Bootstrap 95% confidence intervals for spatial structure indices in the high-density class (n = 5, 1000 iterations).

Comprehensive evaluation indicators for spatial structure	Mean	Bootstrap SE	95% CI (lower)	95% CI (upper)	CV
SPV	0.1984	0.0121	0.1764	0.2221	0.137

Comprehensive evaluation indicators for spatial structure	Mean	Bootstrap SE	95% CI (lower)	95% CI (upper)	CV
Q	0.3328	0.0145	0.3049	0.3606	0.096
FSI	1.2347	0.0198	1.1964	1.2758	0.036
CDEV	0.2877	0.0088	0.2709	0.3055	0.068
CAPV	0.3442	0.0117	0.3218	0.3678	0.076

Note: Bootstrap resampling was performed with 1000 iterations. CI, confidence interval; CV, coefficient of variation (SD/mean). CV < 0.14 for all indices indicates stable mean estimates. Note: Histograms,1000 bootstrap means; Blue dashed: original mean. Green dotted: 95% CI. Upper-right: mean, 95% CI, and CV. Narrow distributions and CV < 0.14 confirm reliable estimates. SPV, Q, FSI, CDEV and CAPV are as defined in Table 11.

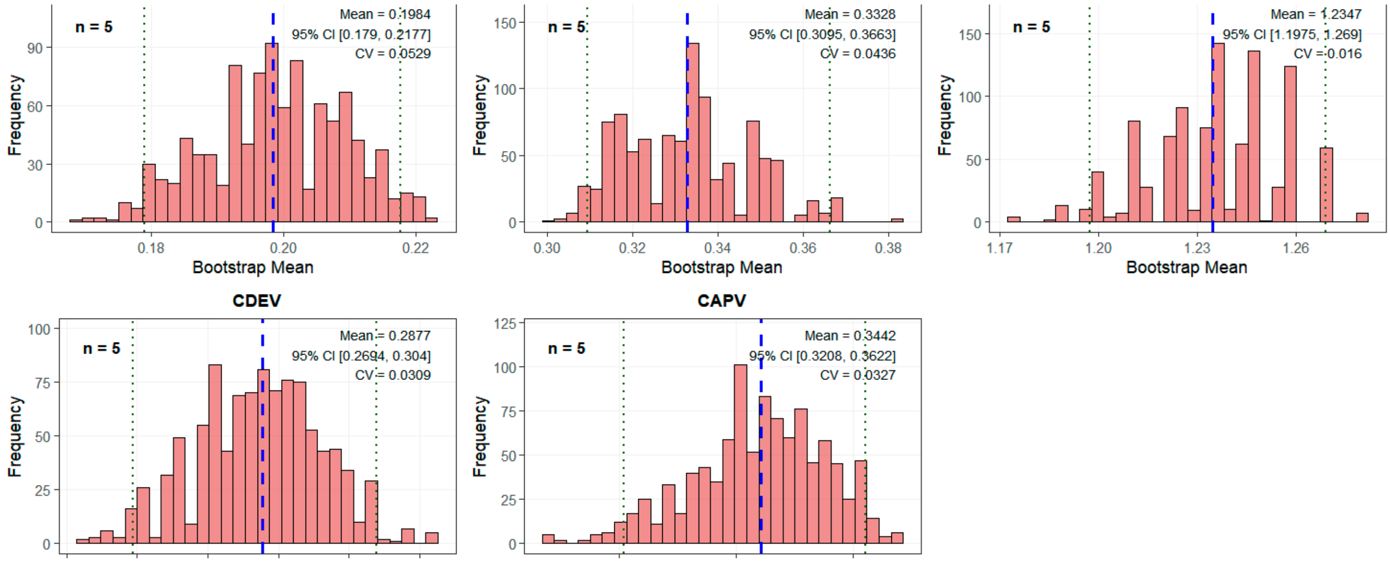

Supplementary Figure S1. Bootstrap 95% confidence intervals for spatial structure indices in the high-density class (n = 5) (Blue dashed line: original mean. Green dotted lines: 95% confidence interval. Upper-right corner: mean, 95% CI, and coefficient of variation (CV). Narrow distributions and low CVs (all < 0.14) indicate reliable mean estimates despite the small sample size). Note: SPV, Q, FSI, CDEV and CAPV are as defined in Table 11.
